# Treatment of severe metformin-associated lactic acidosis with renal replacement therapy and tris-hydroxymethyl aminomethane: a case report

**DOI:** 10.1186/s13256-023-04201-8

**Published:** 2023-10-20

**Authors:** Diana Yusim, Bogdan Tiru, Marat Abdullin, Daniel L. Landry, Spencer Hodgins, Gregory L. Braden

**Affiliations:** 1 Department of Medicine, UMass Chan Medical School-Baystate, Springfield, MA USA; 2 Division of Critical Care Medicine, UMass Chan Medical School-Baystate, Springfield, MA USA; 3 Division of Nephrology, UMass Chan Medical School-Baystate, 759 Chestnut Street, Springfield, MA 01199 USA

**Keywords:** Metformin-associated lactic acidosis, Tris-hydroxymethyl aminomethane, Renal replacement therapy, Type B lactic acidosis

## Abstract

**Background:**

Type B lactic acidosis is a rare but serious side effect of metformin use. The risk of metformin-associated lactic acidosis is elevated in renal or liver impairment, heart failure and in metformin overdose. Metformin-associated lactic acidosis is treated with renal replacement therapy although this can be limited by metformin’s large volume of distribution and a patient’s hemodynamic instability. Tris-hydroxymethyl aminomethane is a buffer that rapidly equilibrates in liver cells and increases the intracellular pH of hepatocytes. Intracellular alkalosis increases lactate uptake by the liver and can promote gluconeogenesis which results in increased lactate metabolism and decreased lactate production. Unlike intravenous bicarbonate which can worsen acidosis due to carbon dioxide retention and hypocalcemia, tris-hydroxymethyl aminomethane does not generate large amounts of carbon dioxide and can improve cardiac contractility in experimental models.

**Case presentation:**

We present a case of a 43-year-old African American male who intentionally ingested 480,000 g of metformin. He developed severe metformin-associated lactic acidosis that was refractory to 21 hours of high flux hemodialysis. This was followed by an additional 12 hours of high flux hemodialysis augmented by continuous intravenous infusion of tris-hydroxymethyl aminomethane. After initiating tris-hydroxymethyl aminomethane, the patient had rapid reversal of lactic acidosis and was weaned off vasopressors and mechanical ventilation.

**Conclusions:**

While metformin-associated lactic acidosis can be treated with renal replacement therapy, severe cases of lactic acidosis may not be amenable to renal replacement therapy alone. Through its unique buffer mechanisms, tris-hydroxymethyl aminomethane can be used in conjunction with dialysis to rapidly improve acidosis associated with metformin.

## Background

Metformin-associated lactic acidosis (MALA) is rare, with an estimated incidence of 6 cases per 100,000 patient-years but is associated with high mortality, averaging 50% [1, 2]. MALA is typically defined by a metformin blood concentration greater than 5 mmol/L and arterial pH less than 7.35 in association with metformin exposure [1]. Severe lactic acidosis is usually found in a patient receiving metformin despite major contraindications, such as chronic kidney disease, congestive heart failure, liver impairment, but it can also be due to voluntary overdose and intoxication [3, 4].

The intestine and the liver are the main sources of metformin-related lactate production [5]. In experimental animal models, metformin increases splanchnic lactate production by stimulating anaerobic glucose metabolism [5]. In hepatocytes, metformin suppresses gluconeogenesis, which impairs the conversion of lactate to pyruvate [6]. Proposed mechanisms include inhibition of pyruvate carboxylase and suppression of mitochondrial complex I through inhibition of glycerophosphate dehydrogenase [2, 6].

The initial therapy for MALA is resuscitation and supportive care with intermittent hemodialysis or continuous renal replacement therapy (CRRT) [1]. Renal replacement therapy (RRT) is recommended in cases of severe metformin poisoning where lactate concentration is > 20, or blood pH is ≤ 7.0 [1]. Dialysis enhances metformin elimination, treats metabolic acidosis, improves hyperlactatemia, and supports kidney function [1]. Sodium bicarbonate administration may be harmful because it can paradoxically worsen acidosis and impair myocardial contractility [7].

As opposed to sodium bicarbonate, tris-hydroxymethyl aminomethane (THAM) is a buffer that may be an effective therapy for MALA when used in conjunction with RRT. THAM is a biologically inert amino alcohol that supplements the buffering capacity of the blood bicarbonate system, accepting a proton, generating bicarbonate, and decreasing the partial pressure of carbon dioxide in arterial blood [8]. It rapidly distributes through the extracellular space and slowly penetrates the intracellular space, except in erythrocytes and hepatocytes where there is complete uptake within minutes [8]. By increasing intracellular pH of hepatocytes, THAM can increase liver lactate metabolism and decrease lactic acid production, potentially reversing the acidosis induced by metformin.

Our case describes the successful use of THAM to augment renal replacement therapy in a patient with severe MALA complicated by oliguric renal failure. We emphasize the role of THAM with concomitant RRT for the rapid correction of acidemia secondary to metformin toxicity.

## Case presentation

A 43-year-old African American male with a past medical history of type II diabetes mellitus, dyslipidemia, non-alcoholic steatohepatitis, major depressive disorder, and tobacco dependence was hospitalized after a suicide attempt by ingestion of 480,000 mg of metformin. He presented with altered mental status and continuous vomiting. Initial vital signs showed a blood pressure of 90/52 mmHg, pulse rate of 50 beats per minute (bpm), respiratory rate of 40 breaths per minute. His point of care glucose was 100 mg/dL. He received several isotonic intravenous fluid boluses with 0.9% saline, was intubated for airway support, and received activated charcoal and polyethylene glycol via a nasogastric tube. The initial serum potassium was 4.9 mmol/L, blood urea nitrogen (BUN) was 10 mg/dL, creatinine was 1.2 mg/dL, bicarbonate (HCO_3_) was 16 mmol/L, anion gap was 22, serum lactate was 6.6 mmol/L, and arterial pH was 7.16. Labs 4 hours later showed serum potassium of 7.4 mmol/L, HCO_3_ of 12 mmol/L, and lactate of 11.8 mmol/L. Given the amount of ingestion, worsening lactic acidosis, and hyperkalemia, emergent high flux hemodialysis (HFHD) was initiated with an Optiflux 200 (Fresenius Medical Care, Waltham, MA, USA) on a Gambro Phoenix Hemodialysis System (Gambro, Deerfield, IL, USA) with a blood flow of 450 mL/minute, dialysate flow of 500 mL/minute, potassium 1.0 bath, and a bicarbonate bath of 36 mmol/L.

**Fig. 1:**
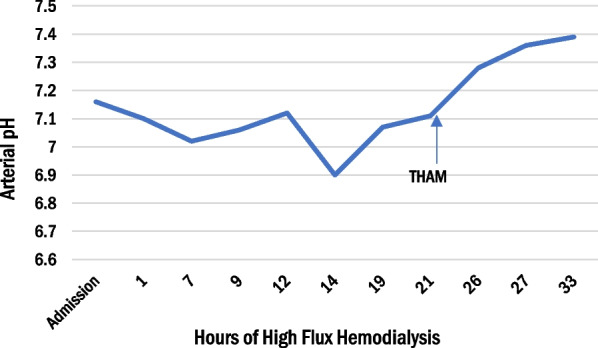
Change in arterial pH, lactate concentration, and bicarbonate with 33 hours of high flux hemodialysis and 12 hours of intravenous tris-hydroxymethyl aminomethane infusion. After initiating THAM, the patient’s metabolic acidosis improved rapidly. Arrow denoted the time of initiation of THAM

**Fig. 2:**
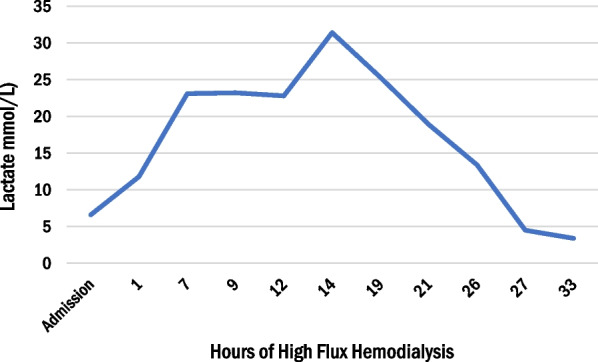
Change in arterial pH, lactate concentration, and bicarbonate with 33 hours of high flux hemodialysis and 12 hours of intravenous tris-hydroxymethyl aminomethane infusion. After initiating THAM, the patient’s metabolic acidosis improved rapidly

**Fig. 3:**
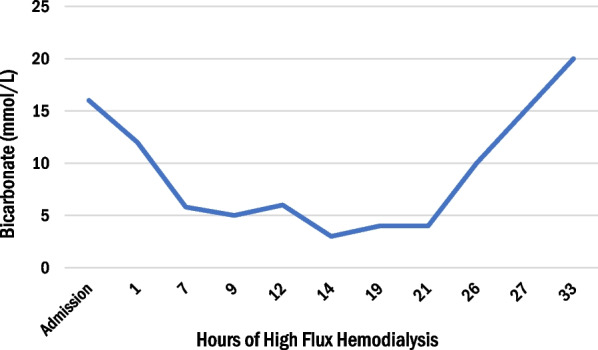
Change in arterial pH, lactate concentration, and bicarbonate with 33 hours of high flux hemodialysis and 12 hours of intravenous tris-hydroxymethyl aminomethane infusion. After initiating tris-hydroxymethyl aminomethane, the patient’s metabolic acidosis improved rapidly

After 12 hours of dialysis, the metformin dialyzer clearance was 416 mL/minute, which is significantly better than earlier reports of 68–228 mL/minute [1]. Labs at that time were significant for pH 7.12, bicarbonate 7, and lactate of 22.8 mmol/L (arterial blood gas). As the acidemia reached a critical level, the patient began to develop worsening hypotension and required initiation of norepinephrine and vasopressin. Dialysis was continued for an additional 8 hours after a break of 1 h. Due to the desire for rapid metformin clearance, intermittent hemodialysis (HD) was preferred over continuous hemodialysis. The next day, 21 hours after HFHD, the serum pH was 7.11, bicarbonate was 4 mmol/L, and lactate was 18.9 mmol/L (shown in Figs. 1, 2, 3). Due to persistent lactic acidosis despite ongoing HD, THAM was chosen to augment the effect of HFHD.

The dose of THAM was calculated as follows: THAM (mL of 0.3 mol/L solution) = lean body weight (kg) × base deficit (mmol/L) [8]. We chose three times the usual base deficit to increase serum HCO_3_ by 15 mmol/L rather than the usual five. We gave 1280 mL of THAM over 12 hours along with HFHD, enough to increase the serum HCO3 by 15 mmol/L. Due to hypoglycemia associated with THAM, the patient was also placed on a D10% dextrose infusion. After a total of 33 hours of HFHD, which included 12 hours of THAM infusion, the serum lactate corrected dramatically from 18.9 mmol/L to 4.5 mmol/L (shown in Fig. 2) and the serum bicarbonate improved from 4 mmol/L to 20 mmol/L (shown in Fig. 3). In fact, over the next 24 hours, the serum lactate normalized along with pH and bicarbonate levels. His pressor support was rapidly weaned off after THAM. The patient also underwent an upper endoscopy for coffee ground emesis, which revealed extensive necrosis with mucosal sloughing of the stomach and duodenum.

During the hospitalization, the patient developed hypervolemia and oliguric acute kidney injury secondary to presumed ischemic acute tubular necrosis with a peak creatinine of 8.6 mg/dL. After failing a trial of 120 mg intravenous furosemide, he was transitioned to continuous veno–venous hemodiafiltration (CVVHDF) for volume and solute management. Over the following days, the patient’s volume status improved, and he was extubated successfully. Due to slow return of renal function, he was placed on a protracted regimen of hemodialysis 3 days a week. By the time of discharge (hospital day 35), the patient’s urine output improved, the serum creatinine normalized to baseline of 0.8 mg/dL, and the patient no longer required hemodialysis.

## Discussion

The severity of lactic acidosis seen in metformin toxicity is exemplified in this patient who developed a blood lactate concentration > 30 mmol/L and arterial pH < 7.0 after metformin overdose. Intermittent HD is a preferred modality for the rapid correction of severe acidemia and removal of metformin and lactic acid compared with CRRT [1]. For example, endogenous metformin clearance can be up to 500 mL/minute in those with intact kidney function [1, 4]. Extracorporeal clearance can exceed 200 mL/minute with intermittent HD and up to 50 mL/minute with CRRT [1]. However, RRT alone can sometimes fail to correct the metabolic acidosis in metformin toxicity. This was seen in our patient who was persistently acidotic after 22 hours of intermittent hemodialysis, despite a calculated metformin dialyzer clearance of 416 mL/minute.

The limitation of RRT can be explained by metformin’s large volume of distribution and two compartment elimination kinetics [9]. Although metformin is readily dialyzable due to its low molecular weight and lack of protein binding, there can be a rebound in metformin plasma levels due to its cellular accumulation [4]. This leads to a biphasic pattern of elimination [4]. In one series, it took an average of 15 hours of conventional intermittent HD to reduce metformin levels to < 20% of predialysis levels [9]. This may explain why our patient’s serum pH and lactate worsened at hour 14 of intermittent HD, despite showing early improvement (Figs. 1, 2).

In cases such as ours where severe MALA is complicated by compromised hemodynamics with volume shifts, hemodialysis alone may provide too slow a correction of metabolic derangements. Given the persistent acidemia and hemodynamic instability, THAM was started to augment RRT. With the introduction of THAM, the patient’s acidosis dramatically improved and normalized (Figs. 1, 2, 3).

The mechanism by which THAM can rectify MALA is complex. We believe that THAM may have quickly increased the intracellular pH of hepatocytes, which would have affected liver lactate metabolism. In animal models of rats, intravenous THAM equilibrates in liver tissue by 3 minutes, but it takes much longer (up to 24 hours) for this to enter skeletal muscle [10]. Intracellular alkalosis can have profound effects on the liver enzymes involved in gluconeogenesis [11]. Pyruvate carboxylase, which is inhibited by metformin and is the first enzyme in the gluconeogenesis pathway, is markedly pH sensitive and its activity increases *in vitro* as pH rises [11, 12]. Additionally, increasing the pH above 7.4 in human liver tissue decreases lactate dehydrogenase (LDH) A activity in converting pyruvate to lactate and increases LDH B activity in converting lactate to pyruvate [13]. By creating a more alkaline environment, THAM cycles pyruvate through the gluconeogenesis pathway which enhances lactate metabolism. In isolated perfused rat liver, induction of alkalosis also significantly increases liver lactate uptake, possibly allowing additional lactate metabolism [14].

THAM may have also played a role in improving the patient’s shock and facilitating withdrawal from vasopressor support. Acidemia directly impairs myocardial contractility, causes arterial vasodilation, and attenuates the responsiveness of heart and vessels to vasoconstrictive effects of catecholamines, especially when the pH is < 7.10–7.20 [7, 9, 15]. The use of intravenous sodium bicarbonate is widely debated. It has been shown to cause carbon dioxide retention and hypocalcemia, which can worsen acidosis and contribute to hemodynamic compromise; its avoidance improves cardiac function and vasopressor response to catecholamine [7]. THAM is associated with little or no carbon dioxide generation [7]. THAM has been shown to restore the myocardial response to catecholamines in dogs and significantly improve the contractility and relaxation of isolated heart muscle impaired by acidemia [8]. This may explain why our patient’s vasopressor requirements decreased after initiating THAM.

## Conclusion

Metformin-associated lactic acidosis is amenable to renal replacement therapy, although several limitations of metformin dialyzability are noted. Given its large volume of distribution, severe cases of MALA may respond inadequately to renal replacement therapy alone. In addition, MALA-associated shock state may be an indication to favor CRRT over intermittent hemodialysis. Use of CRRT can lead to prolonged acidemia due to lower metformin clearance associated with this form of dialysis. In both situations, we believe that THAM can be used in conjunction with RRT to rapidly restore pH and improve a patient’s hemodynamic state.

## Data Availability

All data generated or analyzed during this study are included in this article. Further enquiries can be directed to the corresponding author.

## Abbreviations

CRRT: Continuous renal replacement therapy
CVVHDF: Continuous veno–venous hemodiafiltration
LDH: Lactate dehydrogenase
MALA: Metformin-associated lactic acidosis
RRT: Renal replacement therapy
THAM: Tris-hydroxymethyl aminomethane
HFHD: High flux hemodialysis
